# Emerging human alveolar echinococcosis in Hungary (2003–2018): a retrospective case series analysis from a multi-centre study

**DOI:** 10.1186/s12879-021-05859-5

**Published:** 2021-02-10

**Authors:** Balázs Dezsényi, Zsolt Dubóczki, Tamás Strausz, Eszter Csulak, Veronika Czoma, Zsolt Káposztás, Mária Fehérvári, Áron Somorácz, András Csilek, Attila Oláh, Kálmán Almási, Attila Patonai, Dénes Görög, Zoltán Széll, Zoltán Tolnai, Tamás Sréter, József Danka, Herbert Auer, Beate Grüner, Thomas F. E. Barth, Adriano Casulli

**Affiliations:** 1 Temporarily unaffiliated, Budapest, Hungary; 2 National Institute of Oncology, Tumour Surgery Centre, Budapest, Hungary; 3 National Institute of Oncology, Centre of Surgical and Molecular Tumor Pathology, Budapest, Hungary; 4 Somogy County Teaching Hospital, Department of Surgery, Kaposvár, Hungary; 5 Somogy County Teaching Hospital, Department of Pathology, Kaposvár, Hungary; 6 Jósa András County Teaching Hospital, 4th Department of Internal Medicine, Nyíregyháza, Hungary; 7 2nd Department of Pathology, Semmelweis University, Budapest, Hungary; 8 Borsod-Abaúj-Zemplén County Teaching Hospital, Department of Infectology, Miskolc, Hungary; 9 Petz Aladár County Teaching Hospital, Department of Surgery, Győr, Hungary; 10 Petz Aladár County Teaching Hospital, Department of Pathology, Győr, Hungary; 11 Department of Transplantation and Surgery, Semmelweis University, Budapest, Hungary; 12 National Reference Laboratory for Parasites, Fish and Bee Diseases, National Food Chain Safety Office, Budapest, Hungary; 13 National Reference Laboratory for Human Parasitic Diseases, National Public Health Centre, Budapest, Hungary; 14 Department of Medical Parasitology, Centre of Pathophysiology, Infectology and Immunology, Institute of Specific Prophylaxis and Tropical Medicine, Medical University Vienna, Vienna, Austria; 15 Division of Clinical Infectious Diseases, Department of Internal Medicine III, University Hospital Ulm, Ulm, Germany; 16 Department of Pathology, Ulm University, Ulm, Germany; 17 WHO Collaborating Centre for the epidemiology, detection and control of cystic and alveolar echinococcosis, Department of Infectious Diseases, Istituto Superiore di Sanità, Rome, Italy; 18 European Union Reference Laboratory for Parasites. Department of Infectious Diseases, Istituto Superiore di Sanità, Rome, Italy

**Keywords:** Human alveolar echinococcosis, *Echinococcus multilocularis*, Clinical epidemiology, Case series, Hungary

## Abstract

**Background:**

Human alveolar echinococcosis (AE) caused by *Echinococcus multilocularis* is an underreported, often misdiagnosed and mistreated parasitic disease mainly due to its low incidence. The aim of this study was to describe the epidemiological and clinical characteristics of human AE patients in Hungary for the first time.

**Method:**

Between 2003 and 2018, epidemiological and clinical data of suspected AE patients were collected retrospectively from health database management systems.

**Results:**

This case series included a total of 16 AE patients. The mean age of patients was 53 years (range: 24–78 years). The sex ratio was 1:1. Four patients (25%) revealed no recurrence after radical surgery and adjuvant albendazole (ABZ) therapy. For five patients (31.3%) with unresectable lesions, a stabilization of lesions with ABZ treatment was achieved. In seven patients (43.8%), progression of AE was documented. The mean diagnostic delay was 33 months (range: 1–122 months). Three AE related deaths (fatality rate 18.8%) were recorded.

**Conclusions:**

AE is an emerging infectious disease in Hungary with a high fatality rate since based on our results, almost every fifth AE patient died in the study period. Differential diagnosis and appropriate surgical and medical therapy for AE is an urging challenge for clinicians in Hungary, as well as in some other European countries where *E. multilocularis* is prevalent.

## Background

Human alveolar echinococcosis (AE) is one of the most dangerous and potentially lethal parasitic zoonosis in the temperate and arctic regions of Europe, which is caused by *Echinococcus multilocularis* (Em), a small tapeworm belonging to family Taeniidae [1]. Em develops into its adult form in the small intestine of canid definitive hosts, mainly the red fox (*Vulpes vulpes*) in Europe [2, 3]. To which extent dogs may play a central role in transmitting AE infection to humans in Europe, is still under debate [4, 5]. Infective Em eggs are mainly released into the environment with the feces of infected foxes and ingested by intermediate hosts, mainly small rodents belonging to family Arvicolinae. Oncospheres hatching from eggs penetrate the intestinal mucosa of Arvicolinae and migrate via portal venous and lymphatic circulation into the capillary bed of the main target organ, namely the liver. The oncospheres develop into a larval metacestode, which slowly grows into an infiltrative parasitic tissue. Humans are dead-end intermediate hosts and may become infected, similarly to small rodents, through the ingestion of Em eggs from contaminated environment or matrices. Human AE is a rare disease associated with nonspecific symptoms and a long incubation period of several years. Therefore, main drivers of *Echinococcus* spp. transmission pathways (food-borne, water-borne or hand-to-mouth) and the identification of potential risk factors are still under scientific debate [4–6].

**Fig. 1:**
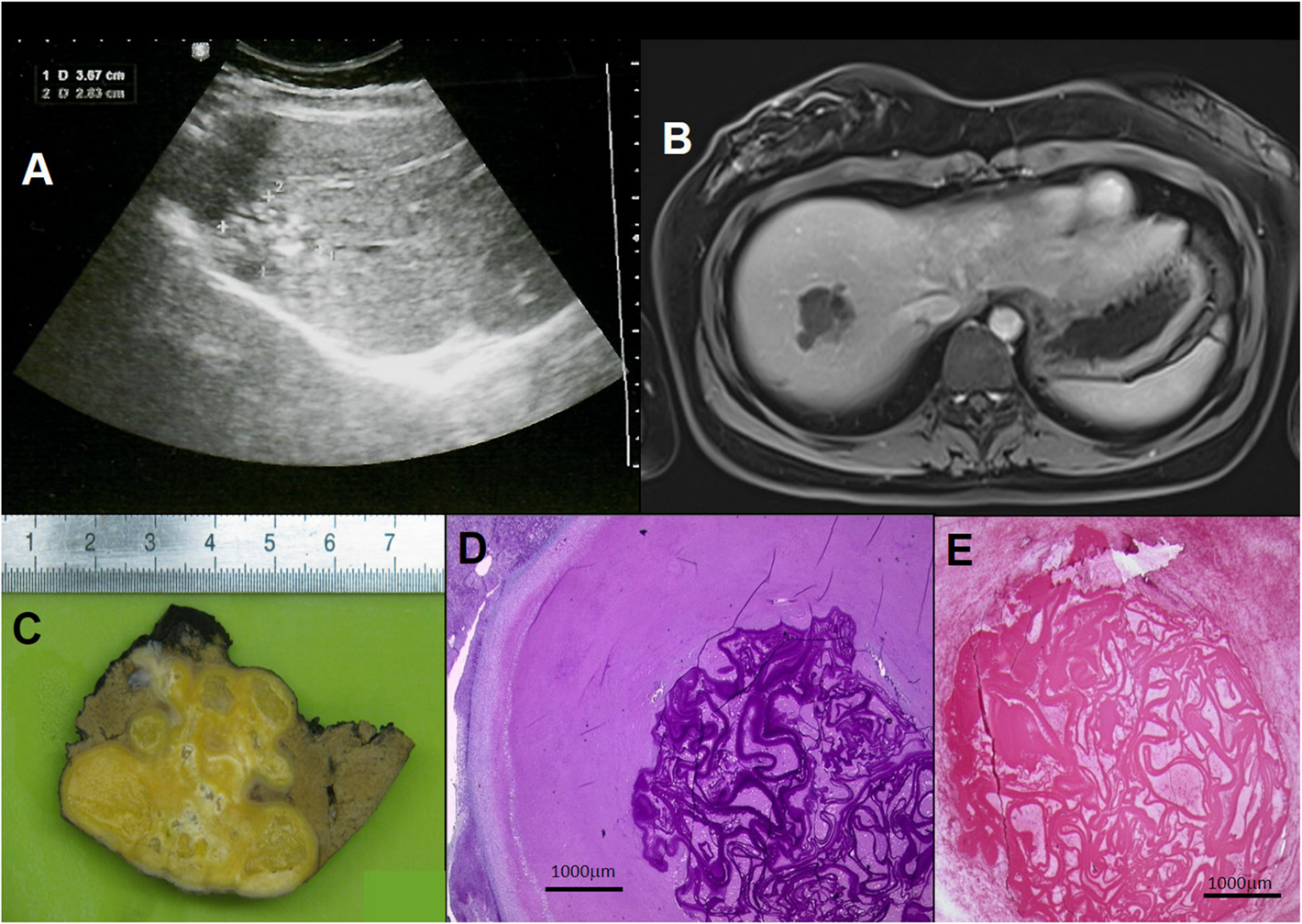
Patient No. 11. **a** Ultrasound image of alveolar echinococcosis (AE) lesion in the liver. **b** Axial T1 weighted MR image of the same AE lesion. **c** Gross picture of the lesion removed by segmentectomy. **d** Microscopic appearance of the lesion: note the multiplex, slender, PAS positive LL of the metacestode surrounded by abundant necrotic material (PAS). **e** Immunohistochemical staining using the monoclonal antibody Em2G11: the antibody marks the laminated layer of Em (LL – red colored band-like structures)

**Fig. 2:**
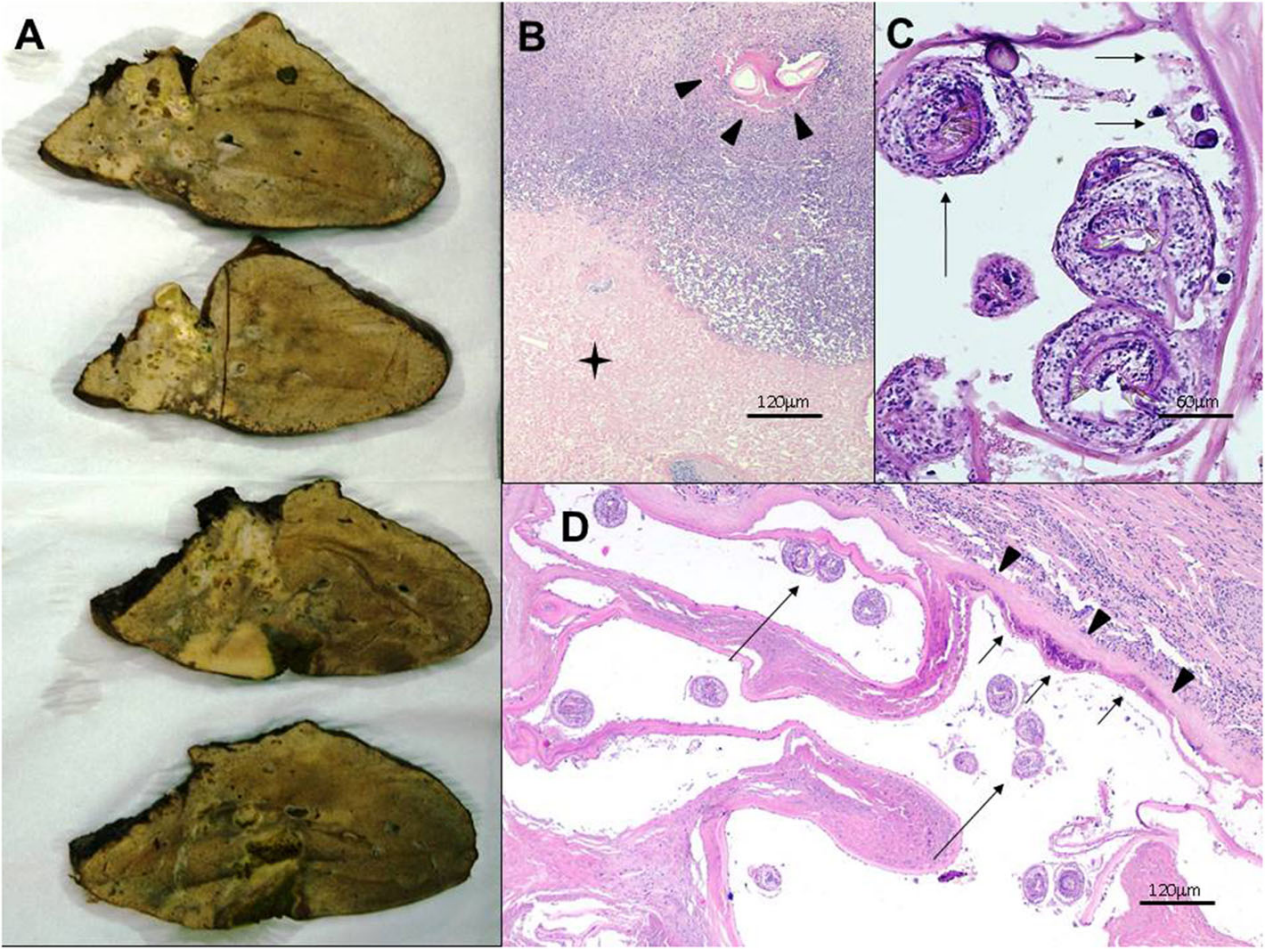
Patient No. 14. **a** Gross view of the resected right lobe of the liver shows heterogeneous solid mass lesion that contains externally budding vesicles and calcified foci with a maximum diameter of a single vesicle around 10 mm. **b**, **c**, **d** Histopathological characteristics of *Echinococcus multilocularis* lesion in the liver. Normal appearance of the liver is distorted. Severe inflammation is present around the necrotic liver tissue (asterisk in **b**). This inflammatory infiltrate consists of lymphocytes and histiocytes. In some areas fragment of cuticular membrane is observed within the necrotic area (arrowheads in **b**). This membrane displays the laminated layer as a tender band-like structure (arrowheads in **d**) with a germinal layer (short arrows in **c** and **d**). Note the invaginated protoscoleces found within the vesicles (long arrows in **c** and **d**)

Clinical management of human AE patients is challenging, even for experts. In humans, oncospheres established in the liver develop as aggregated vesicles (appearing as microcysts at imaging) and then evolving into a tumor-like parasitic mass with locally invasive growth pattern, mimicking malignancy. Histologically, AE-lesions are characterized by necrosis with intermingled fragments of the laminated layer surrounded by a granulomatous inflammation [3] (Fig. 1c; Fig. 2a). Initially, AE is often asymptomatic, and the time from infection to the development of a typical AE-liver lesion is 5 to 15 years in immunocompetent individuals. Diagnosis of AE is based on clinical and epidemiological data, imaging techniques, histopathology and/or nucleic acid detection and serology [7]. Imaging techniques has a central role in the differential diagnosis and clinical management of human AE. In particular, the importance of ultrasonography (US) in hepatic AE rely primarily in the opportunity for early diagnosis [8]. For further characterization of lesions and for investigate extension of AE lesion to adjacent structures and distant metastases, computed tomography (CT) and magnetic resonance imaging (MRI) are useful [7, 9, 10]. During the past decades, several advanced classification systems has been introduced to distinguish types of AE lesions based on different imaging characteristics adjusted for conventional imaging techniques (US, MRI, CT) [8, 11–15]. For interdisciplinary treatment planning, staging based on image findings according the WHO - Informal Working Group on Echinococcosis (WHO-IWGE) PNM classification is recommended [7]. Albendazole (ABZ) is the first drug of choice for medical treatment and is mandatory in all patients [16, 17]. Radical surgery, aiming to completely remove all lesions including satellite (metastatic) lesions followed by a two-year ABZ administration is the standard treatment aiming for cure. The majority of patients are inoperable and need long-term ABZ-treatment [18, 19]. Interventional procedures should be preferred to palliative surgery whenever possible, and radical surgery is the first choice in all cases suitable for total resection of the lesion(s). Rescue liver transplantation is a therapeutic option for AE patients with inoperable lesions and/or chronic liver failure [7, 20].

In this context, our study aims to collect epidemiological and clinical data on all human AE patients diagnosed in Hungary so far by means of retrospective case-series analysis to present the consequences of parasite spreading in previously AE free European countries and provide guidance to clinicians for the management of this emerging helminthic infection in the future.

## Methods

### Case selection and inclusion criteria

A multicentre retrospective case-series analysis was conducted during the study period from 01/01/2003 to 31/12/2018, describing the epidemiological and clinical characteristics of all patients with probable or confirmed diagnosis of AE in Hungary.

The Hungarian patients included in this study were those who had positive serology for Em with one highly sensitive (ELISA or IHA) and one highly specific (Western blot) test and therefore fulfilled the clinical and laboratory diagnostic criteria for probable or confirmed AE patient as proposed by the WHO-IWGE [7]. A probable case was defined as any patient with clinical and epidemiological history, and imaging findings, and positive serology for AE with two positive tests. A confirmed case was defined as the above, *plus* i) histopathology compatible with AE and/or ii) detection of Em nucleic acid sequence(s) in a clinical specimen. Patient(s) who were serologically negative but Em infection was unequivocally confirmed by histopathological methods were also included in this case series.

### Epidemiological and serological data

Baseline demographic and clinical data were collected from medical records from the database management systems of the following centres: National Public Health Centre, National Reference Laboratory for Human Parasitic Diseases, Budapest, Hungary; Central Hospital of Southern Pest National Institute of Hematology and Infectious Diseases, Budapest, Hungary (MedSol); Semmelweis University Department of Transplantation and Surgery, Budapest, Hungary (Medsol); National Institute of Oncology, Budapest, Hungary (MedWorks); Kaposi Mór Teaching Hospital, Kaposvár, Hungary (eMedsol); Borsod-Abaúj-Zemplén County Teaching Hospital, Miskolc, Hungary (Medworks); Petz Aladár County Teaching Hospital, Győr, Hungary (Hospitaly) and Jósa András County Teaching Hospital, Nyíregyháza, Hungary (Helise).

Data regarding potential risk factors were collected by means of questionnaires with dichotomous questions delivered to patients providing informed consent. The following potential risk factors on human infection with Em were investigated: dog ownership, playing with dogs, have a kitchen garden, farming occupation, did hay making in meadows not adjacent to water, went to forests for vocational reasons, ate unwashed strawberries, chewed grass, hunting, handling foxes, consumption of wild vegetables and fruit [4], and presence of foxes at the place of residence.

Serological data were obtained from the Hungarian Reference Laboratory for Human Parasitic Diseases (National Public Health Centre, Budapest, Hungary). Antibody titers were first determined by a high-sensitivity ELISA test (Hydatidosis ELISA, Vircell, Spain; Ridascreen *Echinococcus* IgG, R-Biopharm, Germany) or indirect hemagglutination test (Cellognost-Echinococcosis, Siemens, Germany). As a second high-specificity confirmatory test, Western blot (*Echinococcus* Western blot IgG, Ldbio, France) was applied [21].

### Histopathology, immunohistochemistry, and molecular analysis

Tissue sections from samples included core-biopsies, surgical specimens and specimens from autopsies. Both haematoxylin-eosin (H&E) and Periodic Acid Schiff (PAS) staining were performed. Histological criteria for diagnosis included the identification of multiple cysts, a PAS positive slender (< 1 mm) laminated layer within abundant necrotic areas, a tubular growth pattern accompanied by a granulomatous cell-reaction without any definite fibrous capsule around the lesion [22] (Fig. 1d-e, Fig. 2b-d). Further confirmation derived from an immunohistochemical staining with the monoclonal antibody Em2G11 (Ulm, Germany) [23] (Fig. 1e) or by the detection of Em specific DNA by PCR method (Wien, Austria). Cytological analysis of samples gained by fine-needle aspiration biopsy was also carried out. Cytology alone without further confirmatory tests such as immunocytochemistry was regarded as inappropriate method in diagnosing AE in this case series.

### Clinical data and case follow-up

For detailed clinical analysis, we registered the date (month, year) of the first symptoms and/or findings; the initial symptoms; the first physical and laboratory findings; the initial and final radiomorphological characteristics of AE lesions investigated by conventional imaging methods (US, CT or MRI) with the largest diameter of the lesions in millimeters; the preliminary diagnosis; extrahepatic localization of the parasite at first diagnosis; the date (month, year) of diagnosis of probable or confirmed AE (Table 1). The diagnostic delay in each identified patient as the time period between the first symptoms/findings and the recognition of AE was calculated. Treatment modalities were also analyzed. ABZ was given at a dose of 800 mg per day in two divided doses continuously or intermittently. Duration of ABZ administration were registered in months. In AE patients with resectable parasitic lesion, curative surgical treatment followed the rules of tumor surgery aiming to perform R0 resection without any parasitic residue. Endoscopic and percutaneous interventions (Table 2) and potentially immunosuppressive co-morbidities were also recorded.

**Table 1:** Diagnostic features of human alveolar echinococcosis cohort patients in Hungary (2003–2018)

case no.	1	2	3	4
onset of symptoms or first findings	09.2001	09.2003 focal hepatic lesions during imaging studies	10.2004	08.2008
initial symptoms and physical findings	epigastric pain, vomitus	asymptomatic, hepatomegaly	epigastric and right hypochondriac pain	jaundice, pruritus, right hypochondriac pain
liver function tests: liver enzymes (U/l); sebi (μmol/l)	normal	–	elevated GGT (104)	elevated ALP (1254), GGT (570) and sebi (202)
initial US/CT/MRI (date) radiomorphology largest diameter of AE lesion(s) in mm	US (09.2001) – 15 mm hyperreflective area in SIV CT (03.2003) –echinococcal cysts inboth lobes, number, size, localization unknown	US (04.2005) and CT (08.2009) – 10 typical AE lesions in SIV, SV, SVI, SVIII, 10–30 mm, largest lesion 50 mm	US (10.2004), CT (11.2004) and MRI (06.2005) – one typical AE lesion – 100 mm –in SV, SVI, SVII, SVIII	US (08.2008) – typical central AE lesion −110 mm – in the dichotomy of hepatic common duct, SIV, SV
preliminary diagnosis	echinococcosis	liver tumor, echinococcosis	liver tumor, HCC, liver metastasis	liver tumor, adenocarcinoma
serology Westernblot (Ldbio) P3 Em	positive	positive	positive	positive
core biopsy/surgical sample/autopsy	–	–	core biopsy (2x) surgical sample (1x)	core biopsy during PTC, surgical sample
histopathology/IH/PCR	–	–	histopathology and PCR	histopathology and PCR
type of diagnosis	probable	probable	confirmed	confirmed
month.year of diagnosis	04.2003	04.2004	07.2005	09.2008
latency of diagnosis (in months)	20	8	10	2
extrahepatic localizationat the time of diagnosis	no pulmonary lesion	no pulmonary lesion	peritoneal dissemination no pulmonary lesion	no pulmonary lesion
PNM at diagnosis	PxNxMx	P1N0Mx	P2N1Mx	P3N0Mx
case no.	5	6	7	8
onset of symptoms orfirst findings	2002 asymptomatic hepatic cyst; patient denied investigations	11.2011	12.2012	11.2012
initial symptoms andphysical findings	right hypochondriac pain, vomitus, anasarca, palpable liver tumor (12.2010)	right hypochondriac pain, weightloss, hepatomegaly	asymptomatic, mild hepatomegaly	asymptomatic
liver function tests: liver enzymes (U/l); sebi (μmol/l)	AST (177), ALT (177), GGT (920), ALP (1152), sebi (16)	GGT (105), ALP (543), sebi (7,1)	GGT (335), ALP (999), sebi (7,3)	normal
initial US/CT/MRI (date) radiomorphology largest diameter of AE lesion(s) in mm	US (12.2010) and CT (01.2011) two interconnected pseudocystic AE lesionsin both lobes – 130 mm and 120 mm – dilatated intrahepatic bileducts	US (11.2011) and CT (12.2011) typical AE lesion in SV, SVI −83 mm – and some smaller lesions	CT (04.2013) typical AE lesion in right lobe, 135 mm, periportal biliary and vascular involvement (right v. portae, v. hepatica intermedia)	CT (11.2012) and MRI (08.2014) multiplying small calcified lesions in SV, SVI, SVII, SVIII
preliminary diagnosis	metastasis, tumor, CE	hemangioma, tumor, CE	cholangiocellular carcinoma	liver metastasis
serology Westernblot (Ldbio) P3 Em	positive	equivocal	positive	negative (postoperatively 2x)
core biopsy/surgical sample/autopsy	parasitology and cytology from lesion fluid (FNAB) negative	corebiopsy	corebiopsy	corebiopsy and surgical sample
histopathology/IH/PCR	–	histopathology	histopathology	histopathology andIH
type of diagnosis	probable	confirmed	confirmed	confirmed
month.year of diagnosis	03.2011	01.2012	04.2013	10.2014
latency of diagnosis (in months)	111	1	5	24
extrahepatic localizationat the time of diagnosis	no pulmonary lesion	no pulmonary lesion	undignified pulmonary lesions	no
PNM at diagnosis	P4N0Mx	P2N0Mx	P4N0Mx	P1N0M0
case no.	9	10	11	12
onset of symptoms or first findings	10.2013	04.2012	02.2017	03.2017
initial symptoms and physical findings	right hypochondriac pain, urticaria	right hypochondriac pain, hepatomegaly	epigastric pain, vomitus	right hypochondriac pain
liver function tests: liver enzymes (U/l); sebi (μmol/l)	ALP (125), GGT (86)	normal	normal	elevated ALP
initial US/CT/MRI (date) radiomorphology largest diameter of AE lesion(s) in mm	MRI (12.2015) and CT (01.2016) 2 typical AE lesions in the dichotomy of hepatic veins; in SV/SIVB 55 mm; in SVIII/IVA 53 mm	CT (04.2012) and MRI (10.2012) typical AE lesion in SIV 42 mm	US (02.2017), CT (02.2017) and MRI (03.2017) two AE lesions in SVIII 44 mm and in SVII 12 mm	US (05.2017), CT (05.2017) multiplex AE lesions in both lobes, 40 mm
preliminary diagnosis	atypical rare malignancy liver metastasis	hemangioma, adenoma, liver tumor	hemangioma cholangiocellular carcinoma, fibrolamellar carcinoma	liver metastasis, sarcoidosis, granulomatous hepatitis
serology Westernblot (Ldbio) P3 Em	positive	positive	Echinococcus genus P5	positive
core biopsy/surgical sample/autopsy	–	–	(FNAB) and surgical sample	corebiopsy (2x)
histopathology/IH/PCR	–	–	IH	histopathology
type of diagnosis	probable	probable	confirmed	confirmed
month.year of diagnosis	01.2016	06.2016	05.2017	07.2017
latency of diagnosis (in months)	28	50	4	5
extrahepatic localizationat the time of diagnosis	no	no	no	no
PNM at diagnosis	P3N0M0	P1N0M0	P1N0M0	P2N0M0
case no.	13	14	15	16
onset of symptoms orfirst findings	09.2017	09.2016	04.2008	2008
initial symptoms and physical findings	right hypochondriac pain, hepatomegaly	asymptomatic	asymptomatic	right hypochondriac pain
liver function tests: liver enzymes (U/l); sebi (μmol/l)	elevated liver enzymes	GGT (115)	–	–
initial US/CT/MRI (date) radiomorphology largest diameter of AE lesion(s) in mm	US (10.2017), CT (10.2017) typical AE lesion in SV 80 mm	MRI (09.2016) 15 mm wide hypodens area in right lobe, CT (09.2017) and MRI (11.2017) 75 mm typical AE lesion in SV and SVIII, dilatation of intrahepatic bileducts	US (04.2008), CT (07.2008) typical AE lesion in SV – 54 mm –and three small calcified lesions	US (2008) 20 mm hyperechoic liver lesion, CT (10.2016) and MRI (06.2017) 120 mm typical AE lesion in right lobe (SV-VI-VIII)
preliminary diagnosis	liver tumor	cholangiocellular carcinoma, Klatskin tumor	atypical hepatic cyst	hemangioma, cystadenocarcinoma
serology Westernblot (Ldbio) P3 Em	positive	positive	positive	positive
core biopsy/surgical sample/autopsy	-(FNAB 2x)	surgical sample	surgical sample autopsy	surgical sample
histopathology/IH/PCR	–	histopathology	histopathology	histopathology
type of diagnosis	probable	confirmed	confirmed	confirmed
month.year of diagnosis	12.2017	01.2018	05.2018	08.2018
latency of diagnosis (in months)	4	17	122	115 (+ 12)
extrahepatic localizationat the time of diagnosis	no pulmonary lesion	subphrenic abscess, peribiliar vascular invasion, no pulmonary lesion	falciform ligament, no pulmonary lesion	no pulmonary lesion
PNM at diagnosis	P4N0M0	P4N1Mx	P4N1Mx	P4N0Mx

*E.m Echinococcus multilocularis*, *AE* alveolar echinococcosis, *CE* cystic echinococcosis, *v* vena, *d* ductus, *AST* aspartate aminotransferase, *ALT* alanine aminotransferase, *GGT* gamma-glutamyltransferase, *ALP* alkaline phosphatase, *sebi* serum bilirubin, *US* ultrasound, *CT* computer tomography, *MRI* magnetic resonance imaging, *IH* immunohistochemistry using monoclonal antibody mAbEm2G11, *PCR* polymerase chain reaction, *tx* treatment, *EPI* endoscopic and percutaneous interventions, *ERCP* endoscopic retrograde cholangiopancreatography, *PTC* percutaneous transhepatic cholangiography, *PTD* percutaneous transhepatic drainage, *FNAB* fine needle aspiration biopsy, *S* liver segment, *ABZ* albendazole, *HCC* hepatocellular carcinoma

**Table 2:** Therapeutic features of human alveolar echinococcosis cohort patients in Hungary(2003–2018)

case no.	1	2	3	4
antiparasitic drug tx (duration in months)	–	ABZ (3) 10.2004–12.2004	ABZ (162) continuously since 07.2005	ABZ (12) 11.2008 – 01.2009 and 06.2016–03.2017
surgery	–	–	exploration –unresectable	exploration, fenestration, marsupialisation
EPI	–	–	–	PTD (2x), ERCP
follow-up period in months	27	177	162	124
radiomorphology on final control, largest diameter of AE lesion(s) in mm (date)	CT (04.2005) – pseudocystic AE lesion in left lobe 49 mm, two more AE lesions in right lobe, 35 mm and 24 mm	US (09.2014) stabilization	MRI (12.2018) stabilization	US (12.2018) residual cavity in SIV 70 mm, giant biloma in porta hepatis
PNM at final imaging	P1N0Mx	P1NxMx	P2N1Mx	P3N0Mx
complications	elevated GGT (136), ALP (621), sebi (36,3)	–	–	central biliary obstruction, cholangitis, biloma, bile-leaking
outcome	progression, AE unrelated death	stabilization	stabilization	progression
case no.	5	6	7	8
antiparasitic drug tx (duration in months)	–	ABZ (5) 02.2012–07.2012	ABZ (67) continuously since 06.2013	ABZ (24) postoperatively
surgery	marsupialization, drainage	extended right hemihepatectomy	–	segmentectomy
EPI	ERCP – biliary stent implantation, nasobiliary stent	–	–	–
follow-up period in months	9	84	69	51
radiomorphology on final control, largest diameter of AE lesion(s) in mm (date)	US (11.2011) residual cavity 45 mm, atrophy of right lobe	US (02.2018) no recurrence	MRI (10.2018) and US (10.2018), 109 mm	US (07.2018) no recurrence
PNM at final imaging	P4N0Mx	P0N0Mx	P4N0Mx	P0N0M0
complications	central biliary obstruction, bile-leaking, bilio-peritoneal fistula, injury of bileducts during surgical manipulation, cachexia	postoperative peritonitis, haematoma, bile-leaking, Kehr-drainage	v. cava inferior compression	–
outcome	progression, AE related death	no recurrence	stabilization	no recurrence
case no.	9	10	11	12
antiparasitic drug tx (duration in months)	ABZ (3) lowered dose intermittently in 2016, finally ceased	ABZ (30) continuously since 07.2016	ABZ (21) postoperatively	ABZ (3) 09.2017–11.2017
surgery	–	–	segmentectomy	–
EPI	–	–	–	–
follow-up period in months	36	30	20	18
radiomorphology on final control, largest diameter of AE lesion(s) in mm (date)	MRI (10.2018), CT (10.2018) no progression in liver, new pulmonary micronodules (09.2017)	MRI (11.2018) SIV 70 mm, progression	MRI (06.2018), US (10.2018) no recurrence	US (07.2018) AE lesion in left lobe 65 mm, AE lesion in right lobe 44 mm
PNM at final imaging	P3N0Mx	P1N0Mx	P0N0Mx	P2N0Mx
complications	ABZ hepatotoxicity and allergic reactions, undignified pulmonary microlesions	–	–	–
outcome	stabilization	progression	no recurrence	progression
case no.	13	14	15	16
antiparasitic drug tx (duration in months)	ABZ (12) continuously since 01.2018	ABZ (12) continuously since 01.2018	ABZ (3) 06.2018–09.2018	–
surgery	–	right hemihepatectomy, exstirpation of d. choledochus and cholecystectomy, hepaticojejunostomia	explorative laparotomy	right hemihepatectomy, exstirpation of d. choledochus and cholecystectomy, hepaticojejunostomia
EPI	–	ERCP	ERCP (2x), stent implantation (2x)	ERCP, stent implantation, PTD (2x)
follow-up period in months	13	12	5	1
radiomorphology on final control, largest diameter of AE lesion(s) in mm (date)	MRI (10.2018) AE lesion in SV 79 mm	CT (10.2018) no recurrence	US (05.2018) 120 mm AE lesion occupying left lobe, ascites, dilatated intrahepatic bileducts	–
PNM at final imaging	P4N0Mx	P0N1Mx	P4N1Mx	P0N0Mx
complications	thrombosis and parasitic infiltration of right v. portae, compression of d. hepaticus dexter	leukopenia, hairloss, haematoma in residual left lobe (32 mm) and undignified pulmonary microlesions	compression of d. hepaticus communis, peritonitis, cholangiogen sepsis	compression of d. hepaticus communis, abscessus hepatis, liver insufficiency, septic shock
outcome	stabilization	no recurrence	progression, AE related death	progression, AE related death

*E.m Echinococcus multilocularis*, *AE* alveolar echinococcosis, *CE* cystic echinococcosis, *v* vena, *d* ductus, *AST* aspartate aminotransferase, *ALT* alanine aminotransferase, *GGT* gamma-glutamyltransferase, *ALP* alkaline phosphatase, *sebi* serum bilirubin, *US* ultrasound, *CT* computer tomography, *MRI* magnetic resonance imaging, *IH* immunohistochemistry using monoclonal antibody mAbEm2G11, *PCR* polymerase chain reaction, *tx* treatment, *EPI* endoscopic and percutaneous interventions, *ERCP* endoscopic retrograde cholangiopancreatography, *PTC* percutaneous transhepatic cholangiography, *PTD* percutaneous transhepatic drainage, *FNAB* fine needle aspiration biopsy, *S* liver segment, *ABZ* albendazole, *HCC* hepatocellular carcinoma

Clinical follow-up period was determined as the time interval between the date of AE diagnosis and the date of AE related or unrelated death or the endpoint of this study (31/12/2018). In given cases, the WHO-IWGE PNM classification [7] for AE was carried out based on the initial and final imaging during the study period. We registered AE associated complications including both the sequelae of the natural course of the infection and the consequences of pharmacological, surgical and/or endoscopic interventions. With regards to the outcome, course of AE lesions on conventional imaging was assessed as follows: “recurrence/no recurrence” after radical resection regardless of the duration of adjuvant ABZ treatment, “regression” (decreased size or stable size and declining symptoms or disappearance of former complications such as cholestasis, cholangitis), “progression” (increase in size and/or extension to neighboring organs and/or metastases or new clinical complications due to the AE as cholestasis, need for ERCP-intervention, cholangitis), “stabilization” (no change in size of the lesions and no new complications such as cholestasis and cholangitis) [19]. AE related death or AE unrelated death was also used as clinical outcome categories. At the end of the study, assessment of AE lesions was done by conventional imaging and is given as PNM re-staging (Table 1; Table 2).

## Results

### Epidemiological features

Between 2003 and 2018, a total of 16 patients of AE were reported to the National Public Health Centre in Hungary. Based on clinical and laboratory diagnostic criteria (WHO-IWGE expert consensus), 10 were diagnosed as confirmed (62.5%) and six as probable (37.5%) AE patients. The sex ratio was 1:1. The mean age of patients at the time of first symptoms or findings related to AE was 53 years (range: 24–78 years). Patients originated from rural areas (*n* = 7; 43.8%), suburban areas (*n* = 4; 25%), and urban areas (*n* = 5; 31.3%). Data regarding potential risk factors were collected from 12 out of 16 AE patients. The following potential risk factors were recorded in our series: have a kitchen garden (91.7%), went to forests for vocational reasons (83.3%), dog ownership (66.7%), playing with dogs (66.7%), consumption of wild vegetables and fruits (66.7%), recognizing foxes at the place of residence (58.3%), farming occupation (50%), eat unwashed strawberries (50%), did haymaking in meadows not adjacent to water (16.7%), and chewed grass (16.7%).

### Diagnostic features

Five patients (31.3%) were asymptomatic at first examination, and AE lesions were incidental imaging findings. In symptomatic cases, the most frequent clinical signs were epigastric and/or right hypochondriac pain. Hepatomegaly, vomitus, weight loss and pruritus were observed in six patients (37.5%), while palpable liver mass was detected in one patient (6.3%). Further physical signs were urticaria and anasarca. One patient (stage P3N0Mx) had jaundice (sebi 202 μmol/l) at first presentation (6.3%). Nine patients (56.3%) had elevated liver enzymes (ALP and GGT). Imaging studies (US, CT, MRI) revealed typical hepatic AE lesions in 14 patients (87.5%). In one probable patient, two interconnected AE pseudocystic lesions were detected in both lobes with 130 mm and 120 mm in diameter. In one confirmed patient, multiple small calcified lesions were identified [22]. Hepatic localization of the parasite and environmental parasitic infiltration at the time of diagnosis was observed in 15 patients (93.8%). The disease stage was P1 in four (25%), P2 in three (18.8%), P3 in two (12.5%) and P4 in six patients (37.5%). Extrahepatic involvement of neighboring organs (N1) was detected in three patients (18.8%). Subphrenic abscess (*n* = 1; 6.3%), dissemination along falciform ligament (n = 1; 6.3%) and dissemination along omental peritoneum (n = 1; 6.3%) were present. The absence or presence of distant metastasis was completely evaluated only in six patients (37.5%). Distant metastasis was not found either of these patients M0 (*n* = 6; 37.5%). Pulmonary metastasis at the time of diagnosis was excluded in 15 patients (93.8%). Based on radiological findings, the following preliminary diagnoses were made: echinococcosis, cystic echinococcosis, liver metastasis, sarcoidosis, granulomatous hepatitis, haemangioma, hepatocellular adenoma, hepatocellular carcinoma, intrahepatic cholangiocarcinoma, cystic neoplasm of the liver and fibrolamellar carcinoma. Based on histopathological findings, the following preliminary diagnoses were made: granulomatous hepatitis, chronic hepatitis with fibrosis, helminthosis and echinococcosis. In 10 patients confirmed by histology, protoscoleces or hooklets were evident in two patients (20%) (Fig. 2c-d). In four patients (25%), immunohistochemistry (*n* = 2; 12.5%) and polymerase chain reaction (n = 2; 12.5%) were used to confirm diagnosis. *Em* antibodies were detected in 13 out of 16 patients (81.3%). Diagnostic delay ranged from 1 to 122 months (mean diagnostic delay: 33 months) (Table 1).

### Therapeutic features

Thirteen out of 16 AE patients (81.3%) received ABZ. Three patients (18.8%) received no ABZ treatment due to misdiagnosis (Table 2). During the whole study period, five AE patients (31.25%) received a daily dose of 800 mg uninterrupted ABZ treatment from the time of diagnosis because of multiple and/or extended unresectable AE lesion(s). In one patient (6.3%), AE was recognized in an advanced stage (P4N1Mx), and the patient died after 3 months of treatment. One unresectable patient (6.3%) was treated with ABZ for a total of 12 months with long interruptions during his 124 months follow-up period. Three patients (18.8%) with unresectable AE lesion(s) received ABZ treatment for less than 4 months from the time of diagnosis. Causes of ceasing therapy were drug-related hepatotoxicity, allergic reactions, virtual stabilization of AE lesions and propagation of liver lesions (with supposed uneffectivity of ABZ treatment). Four patients (25%) received adjuvant ABZ therapy following removal of AE lesion(s) by radical (R0) liver surgery. Four patients (25%) received an incomplete concomitant ABZ treatment with a total of 5 months duration (Table 2).

Surgery was performed in nine out of 16 AE patients (56.3%). The timely diagnosis of AE, as a major impact on choosing the proper method of surgical intervention, was only confirmed in one out of the nine patients (6.3%). In three patients (18.8%), explorative laparotomy was carried out for diagnostic purposes to assess resectability and gain tissue-sample for histopathological analysis. Unresectability was detected in three patients (18.8%) due to extrahepatic peritoneal dissemination (*n* = 1; 6.3%), central localization compressing ductus hepaticus communis (n = 1; 6.3%) and peritoneal dissemination and also the compression of ductus hepaticus communis (n = 1; 6.3%). Radical resection aiming to excise the entire parasitic lesion with safety margin (R0) were done in five patients (31.3%) as follows: extended right hemihepatectomy with feeding catheter jejunostomy (*n* = 1; 6.3%), right hemihepatectomy with hepaticojejunostomy (*n* = 2; 12.5%) and segmentectomy (n = 2; 12.5%). In two patients (12.5%) fenestration and marsupialisation of AE lesions, as palliative methods were performed.

Endoscopic-retrograde-cholangio-pancreatography was performed in five patients (31.3%) because of AE associated biliary obstruction. Endoscopic biliary stents were placed five times in three patients. In one patient, nasobiliary stent placement was also necessary to facilitate bile passage. A total of four percutaneous transhepatic drainage of AE lesions in two patients were performed. Among the five patients (31.3%) who needed endoscopic and/or percutaneous interventions, the following stages were determined at diagnosis: P4N1Mx (n = 2; 12.5%), P4N0Mx (n = 2; 12.5%); P3N0Mx (n = 1; 6.3%) (Table 1; Table 2).

### Outcome

Clinical follow up period ranged from 1 to 177 months (mean 52.4 months). Because of misdiagnosis, the probable first documented Hungarian AE patient was left untreated. Based on the laboratory findings, AE presumably progressed but lack of pathological investigations did not allow to draw any conclusion on this very first patient. In four patients (25%), no recurrence of AE was detected after radical surgery and concomitant ABZ treatment. In these patients, disease free period from curative surgery to the date of final imaging ranged from 10 to 74 months. Stabilization of AE lesions with continuous ABZ treatment was achieved in three (60%) out of five patients with unresectable lesions. One probable AE patient with multiple hepatic AE lesions received ABZ treatment for only 3 months. Ten years later, no progression was detected despite the lack of continuous ABZ therapy. In one probable and unresectable patient intermittent low dose (2 × 100 mg per day) of ABZ therapy had to be ceased because of drug-related hepatotoxicity and allergic reactions. After 26 months without treatment, hepatic AE lesions stabilized, but pulmonary microlesions emerged. Unfortunately, the patient was permanently supported with hydrocortisone for hypadrenia, which may have influenced the course of AE. Progression of AE during the study period was proved in seven patients (43.8%). In our series, progression was not generally accompanied with PNM upstaging of cases but increasing size of AE lesion(s) and/or worsening clinical condition directly related to AE. In two (33.3%) out of six patients, short-term or interrupted ABZ treatment is a plausible explanation for progression. In one case, the size of AE lesion increased beside adequate continuous ABZ treatment. In this patient, disseminating malignant neuroendocrine tumor with liver metastases was diagnosed and simultaneously treated with a probable liver AE lesion. Malignancy, administration of somatostatin analog sandostatin, targeted radionuclide therapy and classical radiotherapy, may play a role in the course of AE as immunocompromising factors. We registered three AE related deaths (18.8%) in our study. In patient No. 5 (P4N0Mx), giant pseudocystic AE was presumably misdiagnosed as abscessing CE. Endoscopic Retrograde Cholangiopancreatography (ERCP), stent implantation, surgical marsupialisation and drainage were performed. Central biliary obstruction, bilioperitoneal fistula, injury of bile ducts with subsequent bile leaking, complete lack of ABZ treatment, cachexia and advanced age were possible factors contributing to death. In patient No. 15 (P4N1Mx), imaging studies and explorative laparotomy revealed a central unresectable AE lesion (120 mm) occupying the left lobe and compressing the common hepatic duct with ascites and parasitic invasion along the falciform ligament. AE was confirmed by histopathology from the surgical sample. Peritonitis and cholangiogen sepsis were the causes of death in this advanced case. In patient No. 16 (P4N0Mx), imaging studies revealed a 120 mm AE lesion misdiagnosed as cystic echinococcosis, abscess or tumor of the liver occupying the right lobe and compressing the common hepatic duct. ERCP, biliary stent implantation, repeated percutaneous transhepatic drainage and finally right hemihepatectomy were performed with hepaticojejunostomy. Postoperative bleeding, liver failure and septic shock led to the death of the patient. AE was confirmed by histopathology postoperatively from surgical sample. Mean diagnostic delay in the three lethal AE patients was around 10 years (116 months) (Table 1; Table 2).

## Discussion

By the end of the 1980s, the “historic endemic area” where Em was known to occur in foxes was composed by four countries (Austria, France, Germany and Switzerland) from Central and Western Europe [24, 25]. In these countries nowadays, the majority of AE patients have a nearly-normal life expectancy and a good quality of life while treated according to WHO-IWGE guidelines [26–28]. The subsequent increased emergence of Em in European red foxes has been traced back to the increase in fox population size due to antirabies vaccination, change of human attitudes towards foxes and other ecological factors [25]. Examination of foxes performed since 1989 revealed that the European endemic area of Em is much larger than previously assumed, and cases of human AE have been described in countries previously not recognized as endemic. Out of the European “historic endemic area”, confirmed and presumably autochthonous human AE cases were reported in Belarus, Belgium, Croatia, Czech Republic, Lithuania, Latvia, Poland, Romania, Slovenia, Slovakia, The Netherlands and Hungary [29–31]. Regarding countries adjacent to Hungary, the first human AE cases were detected in Romania in 1999 [32, 33], Slovakia in 2004 [34], Slovenia between 2001 and 2005 [35] and Croatia in 2014 [36].

**Fig. 3:**
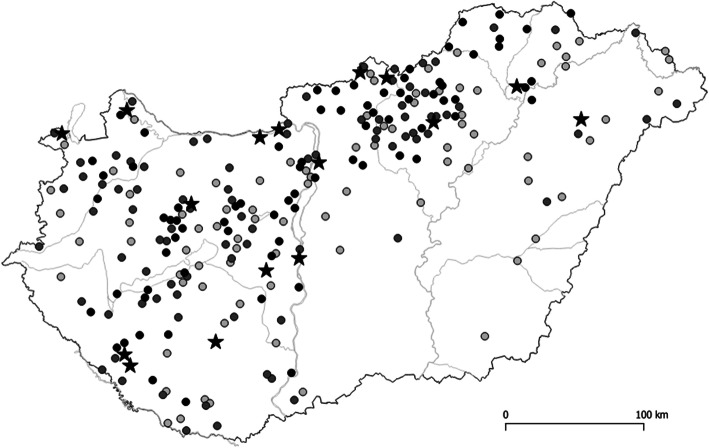
Spatial distribution of 16 AE human cases (stars) and that of 247 red foxes (*Vulpes vulpes*) (dots) infected with *Echinococcus multilocularis* out of 3265 foxes examined between 2008 and 2019. The darkness of the dots reflects the intensity of infection in foxes (light grey: < 10 worms; grey: 1–100 worms; black: > 100 worms) Aapproximately 4% of the fox population of each county was sampled

In Hungary, Em was first recorded in red foxes near to the Hungarian-Slovak border in the Northern Mountain Range in 2002 [37]. After the first confirmation of this parasite in Hungary, further studies were carried out to determine the burden of this parasitic infection in the major wild definitive host, the red fox. Between 2008 and 2019, Em was detected in 18 out of the 19 Hungarian counties, comprising Budapest, with an average prevalence of 7.6% in 3265 analyzed red foxes ([38, 39],unpublished). Highest prevalence was detected in the North-Western half of the country (Fig. 3) [38, 39]. As reported in Fig. 3, the spatial distribution of Em in foxes was highly clustered. This spatial distribution pattern of the parasite can be explained by environmental factors. In fact, the mean annual temperature and the annual precipitations resulted as major statistically significant determinants for the spatial distribution of Em in foxes in Hungary [39]. These results can be attributed to the sensitivity of Em eggs to high temperature and desiccation [40]. Similar relationships with temperature or precipitation and Em infection of foxes or water voles were also observed in France, Germany and Switzerland [41–44]. As a consequence of the fox population increase and change of human behavior, red foxes inhabited urban and suburban areas in Hungary [45] as in other Central European countries [46], increasing the risk of human AE infections. Moreover, golden jackals (*Canis aureus*) have also been identified as definitive hosts in Hungary [47, 48]. Data regarding occurrence of the parasite in intermediate hosts in Hungary are scarce [31]. During a veterinary surveillance study on echinococcosis in livestock conducted between 2015 and 2018, three swine were found to be infected with Em in Hungary, suggesting that swine cases may be regarded as indicators of the environmental contamination by Em eggs [49].

Regarding human echinococcosis in Hungary, cystic echinococcosis (CE) caused by *E. granulosus* sensu *lato* is a well-known parasitic disease since the nineteenth century. In fact, CE is the most prevalent reportable zoonotic helminthosis in Hungary, which is endemic in several regions with considerable disease burden [50]. In contrast, despite the prevalence of Em in foxes, the first confirmed human AE patient in Hungary was reported only in 2008 [51], and the first confirmed autochthonous human AE case was described in 2016 [22]. Herein we reported further 14 AE cases. The limitation of our study may rely on sampling bias, possibly underestimating AE cases. In fact, vast majority of our case series (94%) were collected from the official registry of National Public Health Centre, which is only based on seropositivity for Em. Inadequacies in the reporting system [50] have resulted in the lack of reporting of seronegative AE cases, which might had been diagnosed exclusively by histopathology. Therefore, we cannot exclude that the true number of AE cases might have been higher in Hungary in the past decade. A matter of debate whether the emergence of human AE in Europe is a consequence of the expansion of the classical endemic area through increasing number of fox populations and consequently infected animals or the result of improved awareness and better availability of diagnostic tools. In Hungary, the spread and emergence of Em could be observed in both animals and humans in the past two decades [37–39, 47–49]. Our results showed that the residence of human AE patients overlaps the geographical distribution of Em infected red foxes in Hungary (Fig. 3), suggesting that the majority of human cases may be autochthonous. The majority of AE patients (*n* = 11; 68,8%) originated from rural or suburban areas, which is in line with the observations by other researchers [46] and supporting the hypothesis that living in rural areas may be a proxy for environmental contamination and a driver for AE infection [4]. Regarding other main potential risk factors, almost three quarters of the patients had kitchen garden or visited forests for vocational reasons.

The incidence of human AE is low in Hungary and it is often misdiagnosed as CE, liver abscess or malignancy of the hepatobiliary tract, and most of the patients are only recognized in an advanced and unresectable stage. Six patients (37.5%) were diagnosed with hilar extension of the parasite (P4) and lethality reached 50% in this subgroup. PNM system is useful for AE treatment according WHO-IWGE and assessing prognosis. Retrospective application of PNM has made our results partially comparable on an international level [19]. Unfortunately, ^18^F-FDG-PET/CT, which has found to be useful in the follow-up of AE [52–54], is currently not covered by the health insurance system for patients with non-malignant conditions in Hungary. Furthermore, the lack of complete and detailed CT imaging data did not allow an advanced typing of AE lesions, like EMUC-CT classification [13, 55].

Diagnosis of AE was often delayed (mean diagnostic delay was 33 months in this series) because of misdiagnosis. Hepatobiliary malignancies, especially intrahepatic cholangiocarcinoma (ICC) were frequent misdiagnoses in our AE series. Enhancement pattern and matrix calcifications are the only imaging criteria with a high discriminating power between AE and ICC. During imaging evaluation, the combined presence of no or septal enhancement and calcification ensures 100% specificity for AE [56]. In our series, no or septal enhancement and/or calcification were detectable during preliminary imaging evaluation (CT and MRI) in 11 out of 16 patients (68.8%), which would have allowed the radiologists to distinguish AE from ICC. Non-malignancies such as CE are also pitfalls during differential diagnosis, not allowing further investigations and contributing to diagnostic delay [57]. We emphasize that the awareness of specific imaging characteristics of AE could help to reduce diagnostic delay and accelerate the introduction of antiparasitic drug treatment. In most patients, diagnostic uncertainty or missing histological confirmation led to short-term/interrupted or complete lack of ABZ treatment and to inappropriate surgical interventions, including radical liver surgery with severe complications, which is in line with previous observations made by other researchers [57]. Endoscopic and percutaneous interventions were generally used in defined advanced AE patients. We registered three lethal cases of human AE in our study (fatality rate 18.8%). Since 2005, only three lethal cases caused by autochthonous parasitic infections have been recognized in Hungary, all of them were involved in this series. Misdiagnosis or diagnostic delay may have brought to an inadequate treatment of these three AE cases, resulting in an increased death rate due to palliative surgery or lack of proper medical treatment [58, 59]. In fact, before the advent of medical treatment with benzimidazoles, fatality rate exceeded 90% of AE cases within 10–15 years from diagnosis [60]. The major current causes of death due to AE are either septic shock, liver failure, complications after major liver surgery and complications due to secondary biliary cirrhosis [9, 61–63].

## Conclusions

We conclude that AE is currently the most dangerous human parasitic infection in Hungary. Our results highlight the need of early differential diagnosis supported by accurate imaging evaluation and if serology is not conclusive, additional core-biopsies from progressive liver lesions for histopathological analysis are needed. Histology is a corner stone to distinguish AE from malignancies, bacterial liver abscess and CE, which is also endemic and much more prevalent in Hungary than AE [64]. Early diagnosis and staging of AE allow applying evidence-based treatment methods according to WHO-IWGE and may increase cure rates [28]. ABZ lifelong treatment prevents disease progression and leads to a favorable outcome in most patients. Stage-specific treatment avoids inadequate/palliativeinterventions and potential complications associated with these procedures [61]. The course of AE in immunosuppressed patients needs further observations and precise evaluation [65]. As the results of this study indicate, there is an urgent need for the education of physicians in the diagnosis and clinical management of AE in Hungary and in other newly endemic European countries. This is very important since in historic endemic European countries it has been shown a favorable outcome of AE-patients with adequate treatment.

## Data Availability

The data that support the findings of this study are available at the database management systems of participating study centres but restrictions apply to the availability of these data, which were used under license for the current study and so are not publicly available. Data are however available from the authors upon reasonable request and with permission of the participating institutional ethical boards.

## References

[1] Kern P, Bardonnet K, Renner E, Auer H, Pawlowski Z, Ammann RW (2003). European echinococcosis registry: human alveolar echinococcosis, Europe, 1982-2000. Emerg Infect Dis.

[2] Oksanen A, Siles-Lucas M, Karamon J, Possenti A, Conraths FJ, Romig T (2016). The geographical distribution and prevalence of *Echinococcus multilocularis* in animals in the European Union and adjacent countries: a systematic review and meta-analysis. Parasit Vectors.

[3] Casulli A, Barth TFE, Tamarozzi F (2019). Echinococcus multilocularis. Trends Parasitol.

[4] Conraths FJ, Probst C, Possenti A, Boufana B, Saulle R, La Torre G, Busani L, Casulli A (2017). Potential risk factors associated with human alveolar echinococcosis: systematic review and meta-analysis. PLoS Negl Trop Dis.

[5] Torgerson PR, Robertson LJ, Enemark HL, Foehr J, van der Giessen JWB, Kapel CMO, Klun I, Trevisan C (2020). Source attribution of human echinococcosis: a systematic review and meta-analysis. Plos Negl Trop Dis.

[6] Tamarozzi F, Deplazes P, Casulli A (2020). Reinventing the wheel of *Echinococcus granulosus* sensu lato transmission to humans. Trends Parasitol.

[7] Brunetti E, Kern P, Vuitton DA (2009). Writing panel for the WHO IWGE. Expert consensus for the diagnosis and treatment of cystic and alveolar echinococcosis in humans. Acta Trop.

[8] Kratzer W, Gruener B, Kaltenbach TE, Ansari-Bitzenberger S, Kern P, Fuchs M, Mason RA, Barth TF, Haenle MM, Hillenbrand A, Oeztuerk S, Graeter T (2015). Proposal of an ultrasonographic classification for hepatic alveolar echinococcosis: Echinococcosis multilocularis Ulm classification-ultrasound. World J Gastroenterol.

[9] Bresson-Hadni S, Vuitton DA, Bartholomot B, Heyd B, Godart D, Meyer JP, Hrusovsky S, Becker MC, Mantion G, Lenys D, Miguet JP (2000). A twenty year history of alveolar echinococcosis: analysis of a series of 117 patients from eastern France. Eur J Gastroenterol Hepatol.

[10] Bresson-Hadni S, Delabrousse E, Blagosklonov O, Bartholomot B, Koch S, Miguet JP, Mantion GA, Vuitton DA (2006). Imaging aspects and non-surgical interventional treatment in human alveolar echinococcosis. Parasitol Int.

[11] Reuter S, Nüssle K, Kolokythas O, Haug U, Rieber A, Kern P, Kratzer W (2001). Alveolar liver echinococcosis: a comparative study of three imaging techniques. Infection..

[12] Kodama Y, Fujita N, Shimizu T, Endo H, Nambu T, Sato N, Todo S, Miyasaka K (2003). Alveolar echinococcosis: MR findings in the liver. Radiology..

[13] Graeter T, Kratzer W, Oeztuerk S, Haenle MM, Mason RA, Hillenbrand A, Kull T, Barth TF, Kern P, Gruener B (2016). Proposal of a computed tomography classification for hepatic alveolar echinococcosis. World J Gastroenterol.

[14] Kantarci M, Bayraktutan U, Karabulut N, Aydinli B, Ogul H, Yuce I, Calik M, Eren S, Atamanalp SS, Oto A (2012). Alveolar echinococcosis: spectrum of findings at cross-sectional imaging. Radiographics..

[15] Bulakçı M, Kartal MG, Yılmaz S, Yılmaz E, Yılmaz R, Şahin D, Aşık M, Erol OB (2016). Multimodality imaging in diagnosis and management of alveolar echinococcosis: an update. Diagn Interv Radiol.

[16] Tamarozzi F, Horton J, Muhtarov M, Ramharter M, Siles-Lucas M, Gruener B, Vuitton DA, Bresson-Hadni S, Manciulli T, Brunetti E (2020). A case for adoption of continuous albendazole treatment regimen for human echinococcal infections. Plos Negl Trop Dis.

[17] Siles-Lucas M, Casulli A, Cirilli R, Carmena D (2018). Progress in the pharmacological treatment of human cystic and alveolar echinococcosis: Compounds and therapeutic targets. Plos Negl Trop Dis.

[18] Kern P (2010). Clinical features and treatment of alveolar echinococcosis. Curr Opin Infect Dis.

[19] Kern P, Wen H, Sato N, Vuitton DA, Gruener B, Shao Y (2006). WHO classification of alveolar echinococcosis: principles and application. Parasitol Int.

[20] Koch S, Bresson-Hadni S, Miguet JP, Crumbach JP, Gillet M, Mantion GA, Heyd B, Vuitton DA, Minello A, Kurtz S (2003). European collaborating clinicians. Experience of liver transplantation for incurable alveolar echinococcosis: a 45-case European collaborative report. Transplantation..

[21] Liance M, Janin V, Bresson-Hadni S, Vuitton DA, Houin R, Piarroux R (2000). Immunodiagnosis of *Echinococcus* infections: confirmatory testing and species differentiation by a new commercial Western blot. J Clin Microbiol.

[22] Dezsényi B, Strausz T, Makrai Z, Csomor J, Danka J, Kern P (2017). Autochthonous human alveolar echinococcosis in a Hungarian patient. Infection..

[23] Barth TFE, Herrmann TS, Tappe D, Stark L, Grüner B, Buttenschoen K (2012). Sensitive and specific immunohistochemical diagnosis of human alveolar echinococcosis with the monoclonal antibody Em2G11. Plos Negl Trop Dis.

[24] Eckert J, Deplazes P (1999). Alveolar echinococcosis in humans: the current situation in Central Europe and the need for countermeasures. Parasitol Today.

[25] Schweiger A, Ammann RW, Candinas D, Clavien PA, Eckert J, Gottstein B (2007). Human alveolar echinococcosis after fox population increase, Switzerland. Emerg Infect Dis.

[26] Torgerson PR, Schweiger A, Deplazes P, Pohar M, Reichen J, Ammann RW, Tarr PE, Halkic N, Müllhaupt B (2008). Alveolar echinococcosis: from a deadly disease to a well-controlled infection. Relative survival and economic analysis in Switzerland over the last 35 years. J Hepatol.

[27] Piarroux M, Piarroux R, Giorgi R, Knapp J, Bardonnet K, Sudre B, Watelet J, Dumortier J, Gérard A, Beytout J, Abergel A, Mantion G, Vuitton DA, Bresson-Hadni S (2011). Clinical features and evolution of alveolar echinococcosis in France from 1982 to 2007: results of a survey in 387 patients. J Hepatol.

[28] Grüner B, Kern P, Mayer B, Gräter T, Hillenbrand A, TEF B, Muche R, Henne-Bruns D, Kratzer W, Kern P (2017). Comprehensive diagnosis and treatment of alveolar echinococcosis: A single-center, long-term observational study of 312 patients in Germany. GMS Infect Dis.

[29] Baumann S, Shi R, Liu W, Bao H, Schmidberger J, Kratzer W, Li W, the Interdisciplinary Echinococcosis Working Group Ulm (2019). Worldwide literature on epidemiology of human alveolar echinococcosis: a systematic review of research published in the twenty-first century. Infection.

[30] Vuitton DA, Demonmerot F, Knapp J, Richou C, Grenouillet F, Chauchet A, Vuitton L, Bresson-Hadni S, Millon L (2015). Clinical epidemiology of human AE in Europe. Vet Parasitol.

[31] Deplazes P, Rinaldi L, Alvarez Rojas CA, Torgerson PR, Harandi MF, Romig T, Antolova D, Schurer JM, Lahmar S, Cringoli G, Magambo J, Thompson RC, Jenkins EJ (2017). Global distribution of alveolar and cystic Echinococcosis. Adv Parasitol.

[32] Panaitescu D, Pop M (1999). Alveococoza la om. [Alveococcosis in man]. Rev Rom Parazitol.

[33] Sikó SB, Deplazes P, Ceica C, Tivadar CS, Bogolin I, Popescu S, Cozma V (2011). *Echinococcus multilocularis* in South-Eastern Europe (Romania). Parasitol Res.

[34] Antolova D, Miterpakova M, Radoňak J, Hudačkova D, Szilagyiova M, Začek M (2014). Alveolar echinococcosis in a highly endemic area of northern Slovakia between 2000 and 2013. Euro Surveill.

[35] Logar J, Soba B, Lejko-Zupanc T, Kotar T (2007). Human alveolar echinococcosis in Slovenia. Clin Microbiol Infect.

[36] Dušek D, Vince A, Kurelac I, Papić N, Višković K, Deplazes P, Beck R (2020). Human Alveolar Echinococcosis. Croatia Emerg Infect Dis.

[37] Sréter T, Széll Z, Zs E, Varga I (2003). *Echinococcus multilocularis*: an emerging pathogen in Hungary and Central Eastern Europe?. Emerg Infect Dis.

[38] Casulli A, Széll Z, Pozio E, Sréter T (2010). Spatial distribution and genetic diversity of *Echinococcus multilocularis* in Hungary. Vet Parasitol.

[39] Tolnai Z, Széll Z, Sréter T (2013). Environmental determinants of the spatial distribution of *Echinococcus multilocularis* in Hungary. Vet Parasitol.

[40] Viel JF, Giraudoux P, Abrial V, Bresson-Hadni S (1999). Water vole (*Arvicola terrestris scherman*) density as risk factor for human alveolar echinococcosis. Am J Trop Med Hyg.

[41] Denzin N, Schliephake A, Fröhlich A, Ziller M, Conraths FJ (2014). On the move? *Echinococcus multilocularis* in red foxes of Saxony-Anhalt (Germany). Transbound Emerg Dis.

[42] Fischer I, Graeter T, Kratzer W, Stark K, Schlingeloff P, Schmidberger J (2020). Echinococcosis working group Ulm. Distribution of alveolar echinococcosis according to environmental and geographical factors in Germany, 1992-2018. Acta Trop.

[43] Chauchet A, Grenouillet F, Knapp J, Richou C, Delabrousse E, Dentan C, Millon L, Di Martino V, Contreras R, Deconinck E, Blagosklonov O, Vuitton DA, Bresson-Hadni S (2014). FrancEchino network. Increased incidence and characteristics of alveolar echinococcosis in patients with immunosuppression-associated conditions. Clin Infect Dis.

[44] Burlet P, Deplazes P, Hegglin D (2011). Age, season and spatio-temporal factors affecting the prevalence of *Echinococcus multilocularis* and *Taenia taeniaeformis* in *Arvicola terrestris*. Parasit Vectors.

[45] Szemethy L, Heltai M, Zs B (1999). Effect of oral immunization against rabies on the red fox population in Hungary. Rabies Bull Eur.

[46] Deplazes P, Hegglin D, Gloor S, Romig T (2004). Wilderness in the city: urbanization of *Echinococcus multilocularis*. Trends Parasitol.

[47] Széll Z, Marucci G, Pozio E, Sréter T (2013). *Echinococcus multilocularis* and *Trichinella spiralis* in golden jackals (*Canis aureus*) of Hungary. Vet Parasitol.

[48] Balog T, Nagy G, Halász T, Csányi E, Zomborszky Z, Csivincsik Á (2020). The occurrence of *Echinococcus* spp. in golden jackal (*Canis aureus*) in southwestern Hungary: Should we need to rethink its expansion?. Parasitol Int.

[49] Dán Á, Rónai Z, Széll Z, Sréter T (2018). Prevalence and genetic characterization of *Echinococcus* spp. in cattle, sheep and swine in Hungary. Parasitol Res.

[50] Dezsényi B, Somorácz Á, Danka J, Kucsera I, Barth TFE, Casulli A (2018). Human cystic echinococcosis in Hungary (2000-2014): a retrospective case series analysis from a single-center study. Infection..

[51] Horváth A, Patonai A, Bánhegyi D, Szlávik J, Balázs GY, Görög D (2008). A humán *Echinococcus multilocularis* infectio első hazai esete. Orv Hetil.

[52] Reuter S, Schirrmeister H, Kratzer W, Dreweck C, Reske SN, Kern P (1999). Pericystic metabolic activity in alveolar echinococcosis: assessment and follow-up by positron emission tomography. Clin Infect Dis.

[53] Reuter S, Buck A, Manfras B, Kratzer W, Seitz HM, Darge K, Reske SN, Kern P (2004). Structured treatment interruption in patients with alveolar echinococcosis. Hepatology..

[54] Ammann RW, Stumpe KD, Grimm F, Deplazes P, Huber S, Bertogg K, Fischer DR, Müllhaupt B (2015). Outcome after discontinuing long-term Benzimidazole treatment in 11 patients with non-resectable alveolar Echinococcosis with negative FDG-PET/CT and anti-EmII/3-10 serology. Plos Negl Trop Dis.

[55] Graeter T, Bao H, Delabrousse E, Brumpt E, Shi R, Li W, Jiang Y, Schmidberger J, Kratzer W, Liu W, XUUB consortium (2020). Hepatic alveolar echinococcosis: Comparative computed tomography study between two Chinese and two European centres. Food Waterborne Parasitol.

[56] Mueller J, Stojkovic M, Berger AK, Rosenberger KD, Schlett CL, Kauczor HU, Junghanss T, Weber TF (2016). How to not miss alveolar echinococcosis in hepatic lesions suspicious for cholangiocellular carcinoma. Abdom Radiol (NY).

[57] Stojkovic M, Mickan C, Weber TF, Junghanss T (2015). Pitfalls in diagnosis and treatment of alveolar echinococcosis: a sentinel case series. BMJ Open Gastroenterol.

[58] Buttenschoen K, Gruener B, Carli Buttenschoen D, Reuter S, Henne-Bruns D, Kern P (2009). Palliative operation for the treatment of alveolar echinococcosis. Langenbeck's Arch Surg.

[59] Hillenbrand A, Gruener B, Kratzer W, Kern P, Graeter T, Barth TF, Buttenschoen K, Henne-Bruns D (2017). Impact of safe distance on long-term outcome after surgical therapy of alveolar Echinococcosis. World J Surg.

[60] Wilson JF, Rausch RL, McMahon BJ, Schantz PM (1992). Parasiticidal effect of chemotherapy in alveolar hydatid disease: review of experience with mebendazole and albendazole in Alaskan Eskimos. Clin Infect Dis.

[61] Frei P, Misselwitz B, Prakash MK, Schoepfer AM, Prinz Vavricka BM, Müllhaupt B, Fried M, Lehmann K, Ammann RW, Vavricka SR (2014). Late biliary complications in human alveolar echinococcosis are associated with high mortality. World J Gastroenterol.

[62] Graeter T, Ehing F, Oeztuerk S, Mason RA, Haenle MM, Kratzer W, Seufferlein T, Gruener B (2015). Hepatobiliary complications of alveolar echinococcosis: a long-term follow-up study. World J Gastroenterol.

[63] Ambregna S, Koch S, Sulz MC, Grüner B, Öztürk S, Chevaux JB, Sulima M, de Gottardi A, Napoléon B, Abergel A, Bichard P, Boytchev I, Deprez P, Dumortier J, Frossard JL, Kull E, Meny B, Moradpour D, Prat F, Vanbiervliet G, Thevenot T, Vuitton DA, Bresson-Hadni S, Vuitton L (2017). A European survey of perendoscopic treatment of biliary complications in patients with alveolar echinococcosis. Expert Rev Anti-Infect Ther.

[64] Reinehr M, Micheloud C, Grimm F, Kronenberg PA, Grimm J, Beck A, Nell J, Meyer Zu Schwabedissen C, Furrer E, Müllhaupt B, Barth TFE, Deplazes P, Weber A (2020). Pathology of Echinococcosis: a morphologic and Immunohistochemical study on 138 specimens with focus on the differential diagnosis between cystic and alveolar Echinococcosis. Am J Surg Pathol.

[65] Piarroux M, Gaudart J, Bresson-Hadni S, Bardonnet K, Faucher B, Grenouillet F, Knapp J, Dumortier J, Watelet J, Gerard A, Beytout J, Abergel A, Wallon M, Vuitton DA, Piarroux R (2015). FrancEchino network. Landscape and climatic characteristics associated with human alveolar echinococcosis in France, 1982 to 2007. Euro Surveill.

